# A pre-operative planning framework for global registration of laparoscopic ultrasound to CT images

**DOI:** 10.1007/s11548-018-1799-2

**Published:** 2018-06-02

**Authors:** João Ramalhinho, Maria R. Robu, Stephen Thompson, Kurinchi Gurusamy, Brian Davidson, David Hawkes, Dean Barratt, Matthew J. Clarkson

**Affiliations:** 1 Wellcome/EPSRC Centre for Interventional and Surgical Sciences, University College London, London, UK; 2 Centre For Medical Image Computing, University College London, London, UK; 3 Division of Surgery and Interventional Science, University College London, London, UK

**Keywords:** Laparoscopy, Laparoscopic ultrasound, Surgical planning, Rigid registration, Feature-based registration, Global registration

## Abstract

**Purpose:**

Laparoscopic ultrasound (LUS) enhances the safety of laparoscopic liver resection by enabling real-time imaging of internal structures such as vessels. However, LUS probes can be difficult to use, and many tumours are iso-echoic and hence are not visible. Registration of LUS to a pre-operative CT or MR scan has been proposed as a method of image guidance. However, the field of view of the probe is very small compared to the whole liver, making the registration task challenging and dependent on a very accurate initialisation.

**Methods:**

We propose the use of a subject-specific planning framework that provides information on which anatomical liver regions it is possible to acquire vascular data that is unique enough for a globally optimal initial registration. Vessel-based rigid registration on different areas of the pre-operative CT vascular tree is used in order to evaluate predicted accuracy and reliability.

**Results:**

The planning framework is tested on one porcine subject where we have taken 5 independent sweeps of LUS data from different sections of the liver. Target registration error of vessel branching points was used to measure accuracy. Global registration based on vessel centrelines is applied to the 5 datasets. In 3 out of 5 cases registration is successful and in agreement with the planning. Further tests with a CT scan under abdominal insufflation show that the framework can provide valuable information in all of the 5 cases.

**Conclusions:**

We have introduced a planning framework that can guide the surgeon on how much LUS data to collect in order to provide a reliable globally unique registration without the need for an initial manual alignment. This could potentially improve the usability of these methods in clinic.

## Introduction

Liver cancer is a major health problem and 150,000 patients per year could benefit from liver resection [1]. Laparoscopic liver resection provides benefits over open surgery such as reduced pain and faster recovery for the patient, along with cost savings for the healthcare system due to shorter hospital stays [2]. However, only 5–30% of patients are considered for this approach. In the case where the target tumours are too large or close to critical vascular regions, the procedure is considered high risk given the limited field of view and lack of haptic feedback [3]. Laparoscopic ultrasound (LUS) can potentially decrease this risk since it images sub-surface structures such as vessels and some tumours. However, many tumours are iso-echoic and hence are not visible in the ultrasound images. Therefore, registration of LUS to a pre-operative CT scan using the signal from vascular structures has been proposed as an image-guidance method.

Registration of LUS to CT is a very challenging task because in addition to the liver being deformed during LUS imaging, the imaging field of view is small and restricted by the limited freedom of movement of the LUS probe. The problem then becomes the alignment of a partial subset of the liver to a whole CT scan volume. If the subset is too small and contains for example an individual vessel segment, the registration problem is poorly constrained and depends on a very accurate initialisation. However, our hypothesis is that given enough ultrasound data, a set of vessels and their relative pose and shape is likely to have a unique solution. The task of this paper is to determine what volume needs to be scanned in order to obtain a unique vascular configuration as a function of location across the liver. This information can then be used to guide the surgeon to the appropriate liver volume that needs to be acquired in order to achieve a reliable registration.

### Background

The Ultrasound (US) to CT registration problem has been tackled by several groups. Solutions for freehand US registration have been proposed either by matching US to a combination of the CT signal and a simulated US signal [4], or by registering vessel probability maps from both US and CT [5]. These methods have been demonstrated using images that capture large sections of liver and our preliminary tests suggest such intensity based methods do not work well on the restricted volume captured by the LUS probe. Other authors aligned 3D US instead, using reconstructed vessels as features [6, 7], vessels and liver surface [8, 9] or image intensity gradient information [10]. However, there are currently no 3D probes for laparoscopy on the market.

Few authors have investigated the specific registration of LUS to CT. The first feasibility studies were proposed by Bao et al. in an isolated phantom [11] and Kruecker et al. [12] on a complete experimental setup. The first animal results in an ex-vivo porcine liver were reported by Martens et al. [13] using a surface-based rigid registration with the LapAssistent system. During surgery, besides being moved by breathing motion, the liver is compressed by the LUS probe upon contact and deformed by *pneumoperitoneum* (abdominal insufflation required for laparoscopy). This makes globally rigid solutions such as the three aforementioned ones not sufficient. Even though deformable solutions are available [7, 8, 10, 14, 15], they are hard to validate clinically and may require unfeasible computation times during surgery. Song et al. [1] proposed locally rigid registration to be sufficient given a small enough liver region of interest, and validated a vessel-based approach on in-vivo porcine data. However, the method required manual selection and matching of vessel bifurcations between LUS and CT, a task that is very difficult and time-consuming during surgery. Previously, in [16], we attempted vessel-based registration without establishing landmark correspondences, but the results still depended on the accuracy of the initialisation.

To overcome this operator dependence on initialising the registration, a possible solution is to perform a globally optimised initial alignment. Given the topology of the liver vascular tree, we hypothesise that it is possible to globally align a small LUS volume to the large CT volume if the vasculature captured in the LUS is geometrically unique. The remaining challenge then becomes knowing in which areas of the liver we can find such subsets. Therefore, we propose a pre-operative planning framework to tell the surgeon both where these regions are and how much vascular data in their vicinity is required for a unique alignment.

### Contributions of this paper

To the best of our knowledge, there is currently no method to plan LUS acquisition in terms of registration outcome. Our contributions include:

- A comparison study on the assessment of which liver vascular features are best for registration.
- A framework that predicts how much data must be collected at each region of the liver for a globally optimal registration.
- A proposed global registration approach for LUS to CT data, with results that are compared to the planning.

## Vascular features assessment

In order to understand the nature of the vascular data that is to be used in our planning framework, we perform a preliminary study that compares the registration outcome when using two distinct vascular features: the vessel branching points, also know as bifurcations, and the vessel centreline points.

**Fig. 1:**
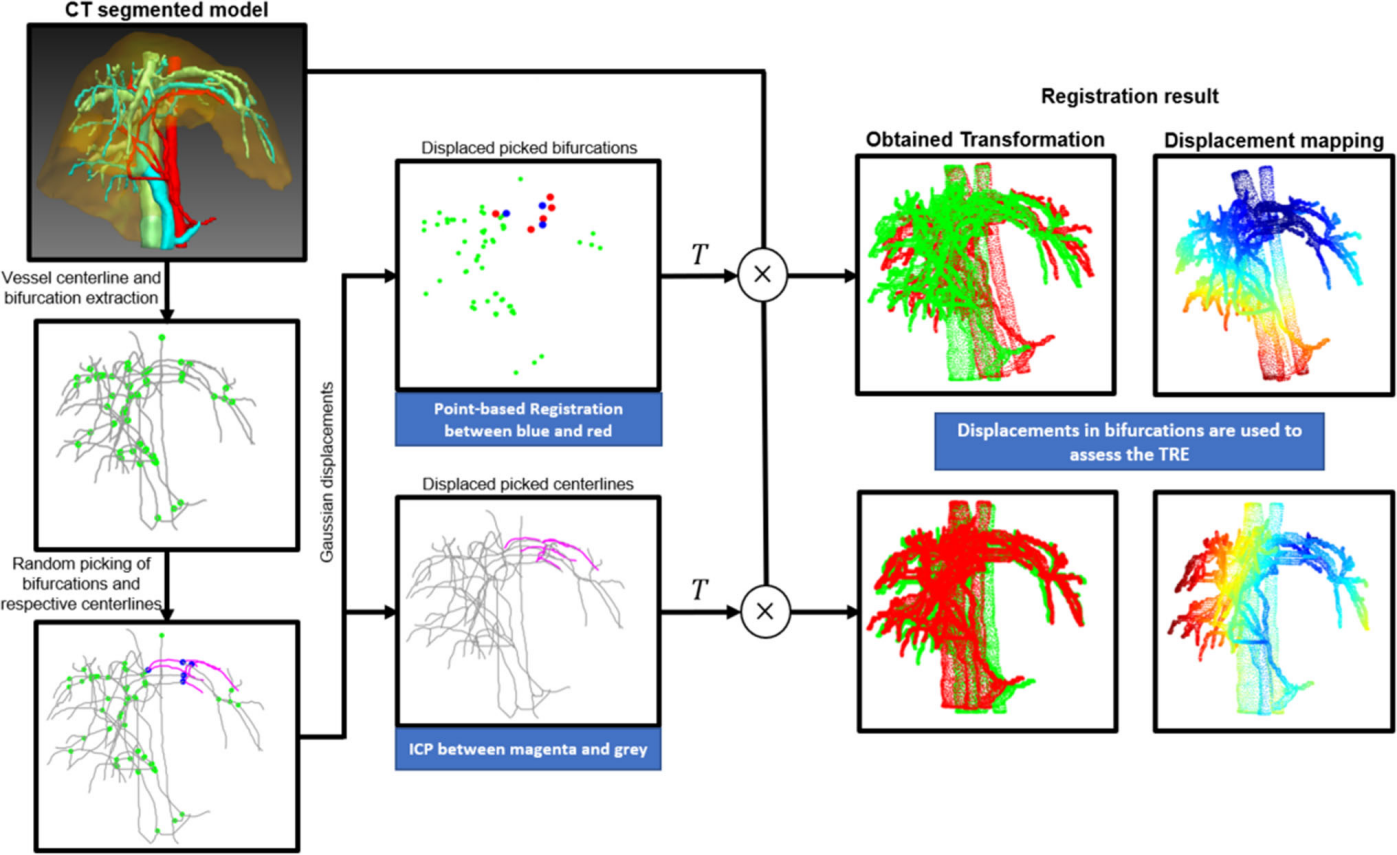
Assessment of registration based on the two different vascular features. Point-based registration on bifurcations and ICP on centrelines are used to recover a transformation *T*. The red vessel models on the right are the resulting configuration, green the original one. Colormaps show predicted displacements between these two, values ranging from blue (low) to red (high)

For this purpose, we simulate the perfect acquisition of subsets of *N* bifurcations and surrounding neighbouring centrelines, apply displacements to simulate expected deformations or segmentation inaccuracies, and register them back to the total model (see Fig. 1). Bifurcations are registered using point-based registration [17], mimicking the scenario where a surgeon picks common landmarks in both modalities and performs a rigid alignment. Centrelines are registered using Iterative-Closest-Point (ICP) [18, 19], simulating the case where correspondences are not known, and the best rigid solution using segmented centrelines is computed. In the case where there are no displacements, the resulting transformation is the identity. Otherwise, a new transformation is obtained and applied to the whole liver model. Using the new bifurcation positions and the original ones, we compute a simulated Target Registration Error (TRE) over all bifurcations and assess the performance of both approaches.

**Fig. 2:**
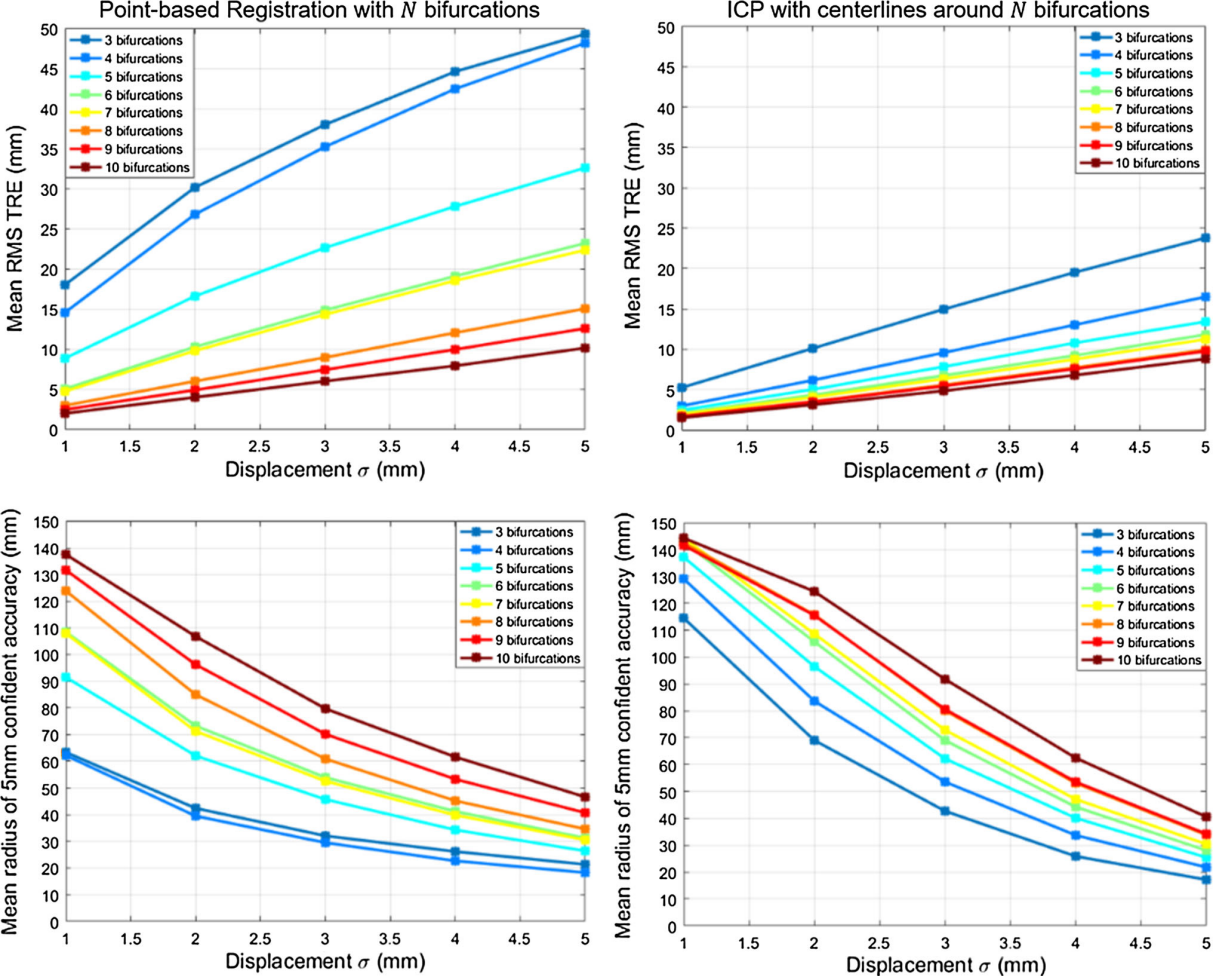
Accuracy results on vessel bifurcations after running results of the first experiment. The top row presents the RMS of the TRE on bifurcations; the bottom row represents the radius where accuracy is better than 5 mm. The left column refers to bifurcation based registration; the right column refers to centreline-based registration

We use this method to test the influence of the number of picked bifurcations and amplitude of applied displacements. 50 combinations of *N* landmarks are randomly selected for *N* ranging from 3 to 10. For each combination, registrations are computed after applying a set of 1000 Gaussian distributed displacements to the selected features, 200 for each deviation $\sigma$ ranging from 1 to 5 mm. In the bifurcation approach, displacements are applied in each point separately. For the centrelines, to better simulate deformation between branches, a random displacement vector is applied to all the points that compose a branch.

The results of this experiment are displayed in Fig. 2. Each curve describes the TRE results for a fixed number of bifurcations given different amplitudes of displacements. In the top row, the Root Mean Square (RMS) of the bifurcation TRE over all the vascular tree is presented in millimetres. Each point in a curve averages a result of 200 registrations over 50 bifurcation configurations. As expected, in both methods the more bifurcations used, the lower the resulting error. Considering 5 mm to be a clinically relevant level of a accuracy, we can see that using point-based registration would require manual identification of 6 bifurcations with an accuracy of 1 mm. This is observed in the green curve with displacement $\sigma =1~\hbox {mm}$. However, to obtain the same result, the ICP approach would only require the centrelines surrounding 3 bifurcations, and without specifying point correspondences. This indicates that within a liver region, fewer vascular branchings are required by the centreline ICP to yield an accurate result. Since in a real scenario LUS registration to CT needs to be accurate in a local region of interest, we also assess the accuracies in terms of a confidence margin. For each registration, we compute the centre of the picked points and find the maximum distance from it below which we capture bifurcations with TRE below 5 mm. In the bottom row of Fig. 2, we present the mean results of this radial distance the same way as in the accuracy plots. In both methods, an increasing number of bifurcations increases this distance. However, unless deviations surpass 3 mm, the confidence margins of ICP are superior to point-based registration: ICP with centrelines around only 3 bifurcations gives a wider mean working radius than point-based registration on 5 bifurcations. Picking the previous example of 6 landmarks and 1 mm of deviation, there is an improvement of 110–150 mm of the working radius. Therefore, besides being more accurate, centreline ICP also requires fewer branching points to be reliable.

These results suggest that up to 6 landmarks would be required for a point-based registration. Clinically, identifying this number of landmarks during surgery would be very challenging and time-consuming for a surgeon, making the vessel centreline approach preferable. We therefore conclude that vessel centrelines will provide a more clinically relevant registration result than bifurcations.

## Methods

Given the demonstration above that centrelines should provide better registration results than bifurcations, our framework aims to assess in a planning stage which centreline point sets are unique and possible to be aligned globally without initialisation. This assessment is performed over the liver surface, guiding a surgeon to acquire the necessary amount of ultrasound data to acquire a unique set of vessels.

**Fig. 3:**
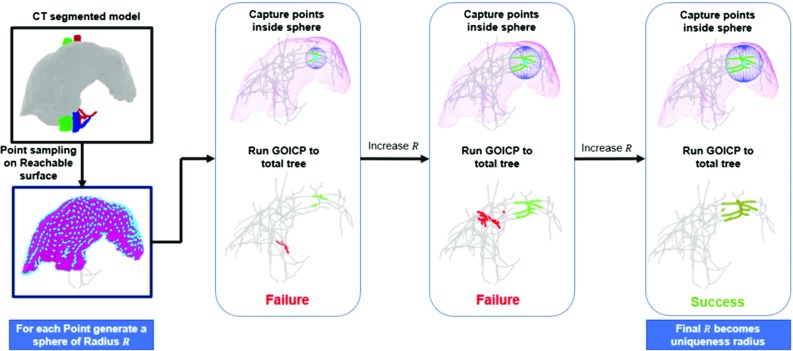
Framework for assessment of uniqueness radius in a surface point of the pre-operative CT. Green represents points captured by a sphere around a point sampled on the pre-operative surface. Red represents GO-ICP resulting alignment

### Uniqueness assessment

Our uniqueness assessment strategy relies on the use of any Global ICP method, such as the Globally Optimal ICP (GO-ICP) proposed by Yang et al. [20]. This algorithm searches the solution space of rotations and translations with a Branch and Bound approach to find the rigid alignment that best minimises the $L^{2}$ norm between two point clouds given a minimum error threshold. We hypothesise that a subset of vessel centreline points is unique if GO-ICP aligns them correctly with the original complete tree using a sufficiently small error threshold. Since the problem is rigid, the global minimum of the distance function is 0, in which the subset is perfectly aligned in the original position. However, by setting the threshold limit to a higher value, we allow GO-ICP to find solutions with larger distance error. When the algorithm reaches this value and outputs a configuration that is not close to the original one, the distance function has at least one local minimum that is not related to the global one, implying that the subset is not geometrically unique.

### Uniqueness planning

Instead of using complete centreline branches as in “Vascular features assessment” section, our framework evaluates the uniqueness of centreline point subsets captured by simulated probe positions along the pre-operative model surface. This process is illustrated in Fig. 3. Firstly, the surface subset that is anterior and visible during surgery is extracted manually and subsampled to generate possible probe contact positions. A sequence of spheres with increasing radius is generated around each of these positions, and the centreline points that are captured in its domain are tested for uniqueness with GO-ICP. We then define the uniqueness radius $R_\mathrm{U}$ as the minimum value at which the captured centreline points are unique. By repeating this process in all the sampled points, a $R_\mathrm{U}$ map is generated which tells the surgeon how broad the acquisition must be in each liver region for a globally unique registration.

### LUS to CT registration

To validate the uniqueness planning, we register continuous sets of 2D tracked LUS images to the pre-operative CT using the same GO-ICP methodology of the previous section. For each set, vessel centrelines are extracted from the images in two steps: firstly, vascular structures are manually delineated in order to obtain binary masks of vessel sections; secondly, centreline points are estimated as the centroids of each of these vessel sections. This segmentation strategy aims to simulate results that could be obtained with an automatic framework using only intensity information from 2D images. Currently, such approaches could be implemented for example using Deep Learning methods. An intra-operative probe contact position *P* of the dataset is computed as the mean of the tracked surface digitised points as defined in [16]. The same expanding radius strategy of “Uniqueness planning” section is then applied to the segmented centrelines using *P* as the sphere centre. In each registration, the TRE between manually picked bifurcations from both the LUS dataset and CT is measured. We define the success radius $R_\mathrm{S}$ as the radius from which the TRE result obtained by GO-ICP reaches a minimum and stabilises. If this minimum is not reached, we consider the registration to be unsuccessful.

**Fig. 4:**
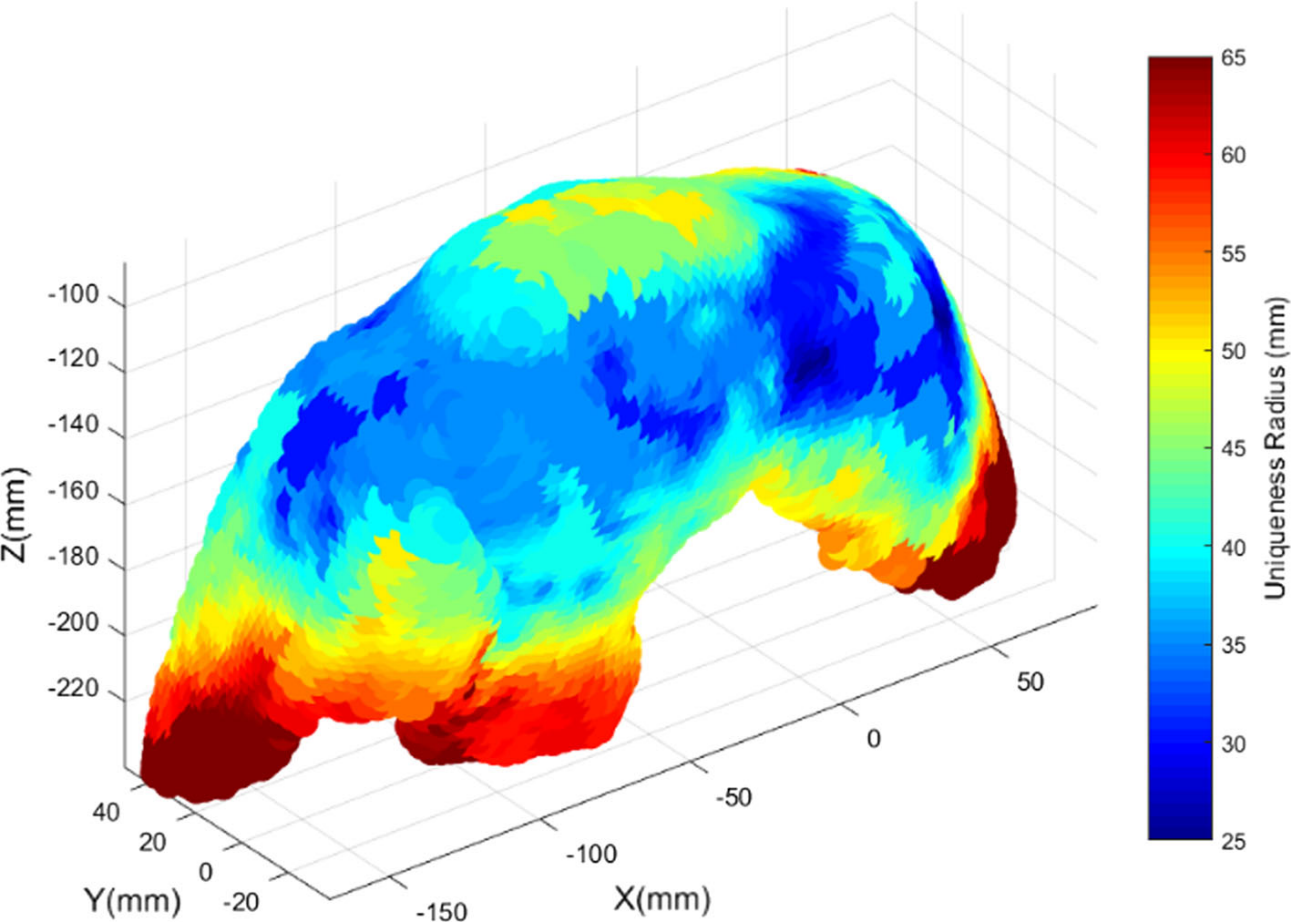
Uniqueness map obtained by sampling 400 points over the visible surface. Sphere radius ranges from 5 to 100 mm in steps of 5 mm. Red and blue represent high and low values of uniqueness radius respectively

In the real LUS data several deformation components are expected. This means that the minimum $L^{2}$ error found by GO-ICP may not be physically correct. To mitigate this effect, we use some prior knowledge of the LUS acquisition to constrain GO-ICP solution search space. For translations, we constrain the dataset to the liver lobe where it was captured, left or right. For rotations, we define a rough estimate of the direction in which the sweep was taken in the CT, and allow only for rotations between $[-\,60^{\circ }, 60^{\circ }]$. This strategy prevents the algorithm from considering physically impossible acquisition configurations.

To compare the result with the planning, we obtain a reference standard transformation by applying point-based registration in the landmarks used for TRE assessment. The aim here is to test how well our uniqueness map predicts the amount of acquired data required for a reliable registration. Therefore, the radius $R_\mathrm{S}$ at which GO-ICP successfully aligns the LUS data to the CT should be similar to the radius $R_\mathrm{U}$ predicted by the framework given the point-based reference standard alignment.

### Experiments

Experiments were performed in retrospective data from a single porcine subject previously acquired in [1]. Surface and vascular models were segmented^1^ from standard abdominal triphase CT scans with resolution $512\times 512\times 2.5~\hbox {mm}$. LUS images of resolution $384\times 456~\hbox {mm}$ were acquired at a rate of 10Hz using an Analogic^2^ SonixMDP and a Vermon^3^ LP7 linear probe which was electromagnetically (EM) tracked by an NDI Aurora^4^ tracking system at a rate of 40Hz.

We test the planning framework by sampling 400 probe contact positions evenly spaced along the surface using the Farthest-point sampling algorithm [21]. We vary the sphere radius in steps of 5 mm in the range [5–100] mm. We set the minimum threshold of GO-ICP to a mean distance error of 2.4 mm. Smaller values give finer results but require larger computation times. We linearly interpolate the sampled surface results to the whole visible surface points to obtain a complete uniqueness radius map.

Global registration experiments are performed in 5 sets of LUS images, 3 acquired from the right lobe of the subject and 2 acquired from the left. In order to reduce fluctuation effects from the EM tracking system, we fit a cubic spline to the digitised surface and apply the result to the centreline points, smoothing them. We run the uniqueness assessment with the same parameters as the planning plus the prior solution space constraints described in “LUS to CT registration” section. Reference standard point-based alignments are obtained separately for each dataset. By applying the resulting transformation to the respective positions *P*, reference probe positions $P_\mathrm{R}$ are obtained and used for comparison with the generated $R_\mathrm{U}$ map.

**Fig. 5:**
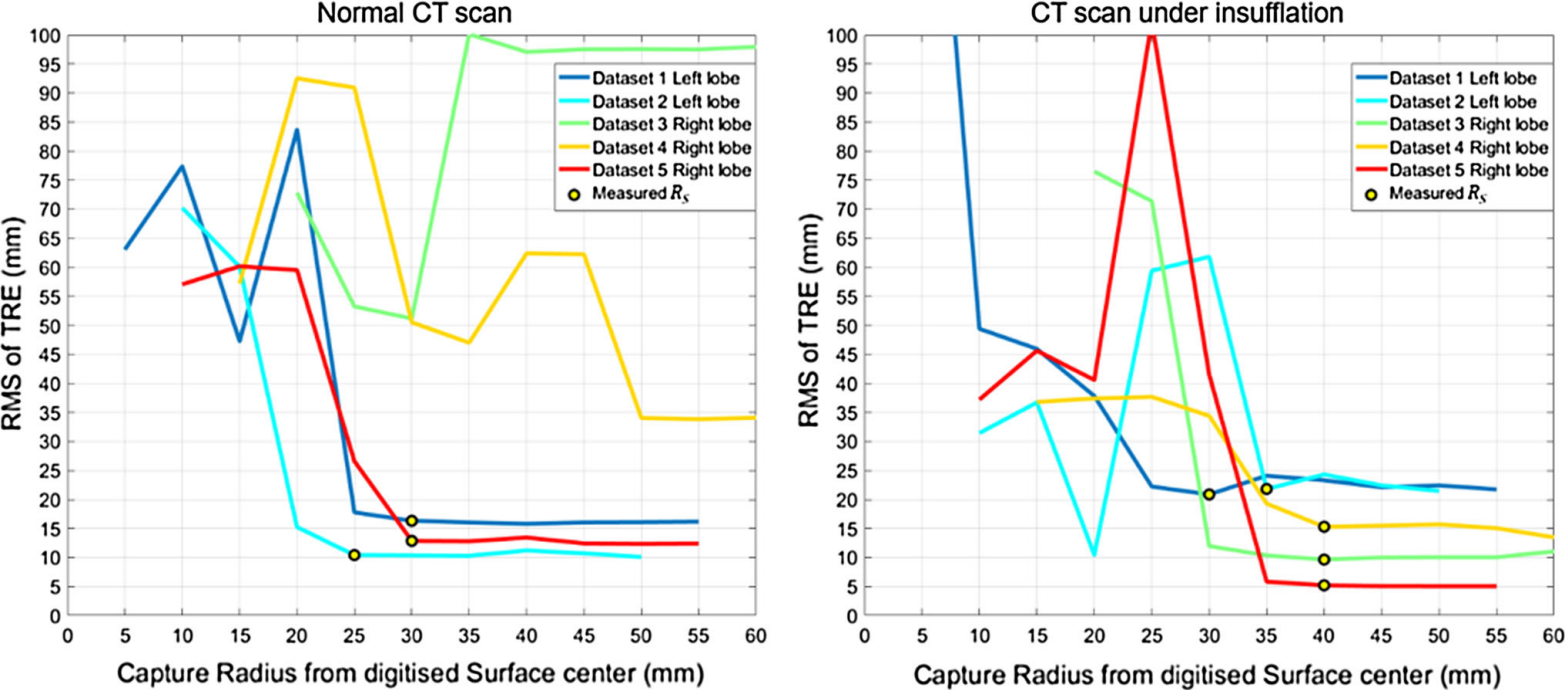
Accuracy results of LUS to CT global registration experiments. Left represents results using a normal CT scan as target; Right represents results using CT scan under insufflation. Yellow markers represent the stable minimum that is measured as $R_\mathrm{S}$

## Results

### Uniqueness planning

Figure 4 shows the obtained uniqueness radius map. Red areas have a high uniqueness radius, implying that broader data collections around them are required for a unique registration. In contrast, blue regions have a lower uniqueness radius and require less data. The highest values are obtained at the liver edges, which is expected given that vasculature is less present in the liver periphery. The best and lowest values are observed in more central regions of the liver, ranging from 25 to 40 mm. The exception is the liver top surface which is more distant from the vasculature, yielding results around 50 mm.

### LUS to CT registration

LUS to CT registration accuracy results are displayed in the left plot of Fig. 5. For each dataset, the RMS of TRE is presented as a function of the capture radius around the respective intra-operative probe contact position. For the cases where TRE stabilises at a minimum and registration is successful, a yellow marker highlights the corresponding success radius $R_\mathrm{S}$. Table 1 summarises the obtained success radii $R_\mathrm{S}$ along with the corresponding $R_\mathrm{U}$ predicted by the planning framework. For each dataset, this $R_\mathrm{U}$ value is taken as the uniqueness radius measured at the planning surface point closest to the reference probe position $P_\mathrm{R}$. Error $E_\mathrm{S}$ represents the RMS of TRE measured at radius $R_\mathrm{S}$. Error $E_\mathrm{R}$ represents the RMS of TRE obtained by the reference standard. Since this standard is the result of direct minimisation of the distance between the landmarks with a rigid solution, $E_\mathrm{R}$ provides the minimum TRE error that can be achieved. It is possible to see that 3 out of 5 cases were successfully registered, and that the respective $R_\mathrm{S}$ values are similar to the prediction $R_\mathrm{U}$, with differences of 1.5, 7.2 and 5 mm. In the two failure cases, registration is never successful, and no comparison with planning can be made. To better understand these cases, we measure $D_\mathrm{R}$, the distance between $P_\mathrm{R}$ and corresponding $R_\mathrm{U}$ planned location. In the successful cases, this distance does not surpass 15 mm. However, in the failure cases, this result surpasses 23 mm, indicating a large difference between intra and pre-operative surface and therefore implying the presence of significant liver tissue deformation.

**Table 1:** Success radius measurement results of LUS datasets

Dataset	Normal CT scan					CT scan under insufflation				
	$R_\mathrm{U}$	$R_\mathrm{S}$	$E_\mathrm{S}$	$E_\mathrm{R}$	$D_\mathrm{R}$	$R_\mathrm{U}$	$R_\mathrm{S}$	$E_\mathrm{S}$	$E_\mathrm{R}$	$D_\mathrm{R}$
1 (Left lobe)	31.5	30.0	16.3	5.9	4.8	39.1	30.0	20.9	8.6	13.2
2 (Left lobe)	32.2	25.0	10.4	1.8	14.8	40.0	35.0	21.8	2.8	2.4
3 (Right lobe)	45.0	–	–	4.7	25.0	43.8	40.0	9.6	6.8	15.8
4 (Right lobe)	40.0	–	–	21.3	23.1	40.5	40.0	15.3	10.3	14.4
5 (Right lobe)	35.0	30.0	12.8	9.4	13.2	45.0	40.0	5.2	3.2	9.1

$E_\mathrm{S}$ represents the RMS of TRE obtained with registration at success radius $R_\mathrm{S}$; $E_\mathrm{R}$ represents the same error for the reference standard transformation; $R_\mathrm{U}$ represents the uniqueness radius predicted by the framework using the reference standard; $D_\mathrm{R}$ represents distance between the reference probe position $P_\mathrm{R}$ and surface location of $R_\mathrm{U}$ measurement. All values are in millimetres

In order to assess how much deformation is influencing this registration problem, we repeat the same planning and registration experiments using an insufflated CT vascular tree and therefore compensate for *pneumoperitoneum*. CT scans under these conditions are not acquired clinically, and hence are not used for planning. However, we present these results for illustrative purposes. Accuracy results of this experiment are presented in the right-hand side of Fig 5. along with the respective radius measurements in the right-hand side of Table 1. In this case, all datasets are registered successfully with a $R_\mathrm{S}$ radius similar to the planning, with differences ranging from 0.5 to 9.1 mm. Furthermore, in agreement with the successful cases of the normal experiments, the distances $D_\mathrm{R}$ never surpass 16 mm.

**Fig. 6:**
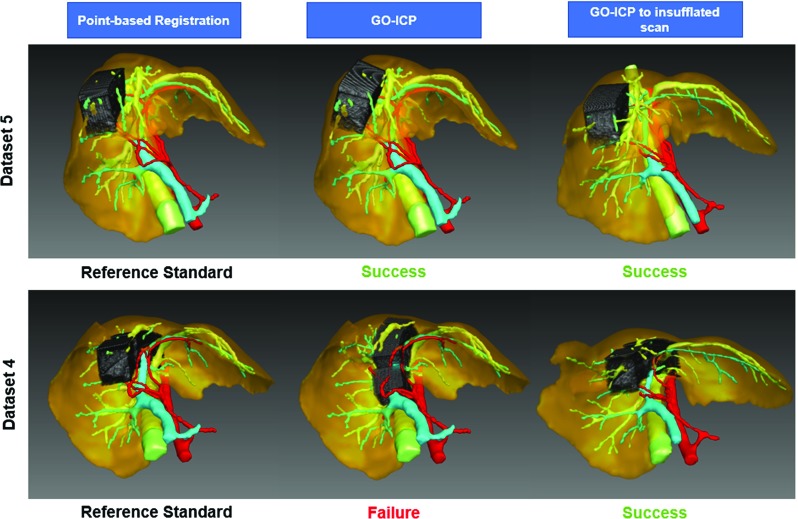
Visual results of registrations of LUS datasets 4 and 5 from the right lobe. Left shows the reference standard results. Middle top shows GO-ICP result with measured success radius; Middle bottom shows GO-ICP registration failure using maximum radius; Right shows GO-ICP result with insufflated scan as target at the measured success radius

Visually, we can observe this improvement in Fig. 6. In each row, the result of each of the three registration experiments is presented. We can observe that for Dataset 5, GO-ICP with the measured $R_\mathrm{S}$ of 30 mm aligns the reconstructed ultrasound sweep in the same region as the reference standard. The same happens if the insufflated scan is used, with the $R_\mathrm{S}$ of 40 mm. In the case of Dataset 4, we observe that registration to the normal scan is not successful with any radius, but with an insufflated scan results improve and a radius $R_\mathrm{S}$ of 40 mm is observed. The same improvement from normal to insufflated scan was obtained with Dataset 3.

## Discussion

The results of our uniqueness mapping are as expected, since surface regions that are far from the vasculature are predicted to require broader data acquisitions. In practical terms, the mapping of Fig. 4 would guide a surgeon to acquire data preferentially in the blue areas, where smaller volumes of data are required for a globally optimal alignment.

In the LUS to CT registration experiments, 3 out of 5 datasets were registered successfully with accuracy values $E_\mathrm{S}$ of 16.3, 10.4 and 12.8 mm (Table 1 column 3). In the two failure cases, large differences between intra-operative and pre-operative surface positions indicate deformation. From the three expected deformation factors listed in “Background” section, surface compression and *pneumoperitoneum* are the ones that may better explain this phenomenon. Further experiments with an insufflated target led to successful registrations with all datasets with accuracies ranging from 5.2 to 21.8 mm. Higher errors above 20 mm and much larger than the reference standard are observed in the left lobe datasets. This may be explained by errors in the manual localisation of bifurcations. Furthermore, retrospective analysis showed that the CT insufflated scan was unreliably segmented in this region. Regardless, the datasets are aligned in the correct region, but with a larger translation error. These results indicate that this registration problem may be more affected by insufflation than surface compression.

In the normal scan experiments, for the 3 successful cases, the planned radius $R_\mathrm{U}$ is similar to the measured success radius $R_\mathrm{S}$ with a maximum difference of 7.2 mm. In the insufflated case, a larger difference of 9.1 mm is observed for dataset 1, a fact which is possibly related to the lower accuracy of the registration. However, it can be observed that the radii $R_\mathrm{S}$ are always lower than the planned $R_\mathrm{U}$. Since we corrected for insufflation in the second experiment, this trend could be related to surface compression—if the surface is pushed closer to the vessels, unique vasculature would be found with a lower capture radius. Regardless, the difference between predictions and measurement is not critical as a surgeon would not be able to collect data with a 5 mm radius precision.

In addition to showing a reasonable agreement with the planning predictions and the obtained results, this approach points to the feasibility of GO-ICP method with prior knowledge for vessel-based registration of LUS to CT. The obtained TRE values obtained in the normal experiment are promising given the fact that we aimed to initially align rigidly a set of vessels in its correct region. Obtaining clinically valuable accuracies below 10 mm could be achieved by compensating for deformation locally afterwards. Using non-optimised code and hardware, the uniqueness map compilation and clinical registration with GO-ICP took 20 h and 10 min respectively. Since the former is a pre-operative off-line step, this high value would not be critical to clinical application.

## Conclusions

We have developed a pre-operative planning framework to guide the surgeon in acquiring LUS data in a way that a reliable registration to CT can be obtained without an initial manual alignment. We consider this framework to be relevant since LUS is particularly difficult to handle, and providing guidance beforehand could increase the use of this registration method and enhance the safety of laparoscopic liver resection.

The major limitation of this work is the restriction of the study to one subject. To mitigate this risk, we show variability by applying our method on data from several different regions of the liver. Another limitation maybe the fact that we do not compensate for any deformation in the framework, leading to registration failures. However, for the purpose of a globally optimal initial alignment, it is difficult to introduce deformation parameters. Since successful registrations were achieved with the insufflated scan, rigid may be sufficient if insufflation is modelled in the pre-operative CT, as in [22].

Our future work directions are twofold. From a technical point of view, we intend to simulate insufflation in our framework and in a later stage probe compression. In terms of validation, we aim to apply this framework in human data in order to obtain clinically translatable results.

## Footnotes

1 www.visiblepatient.com.

2 www.analogicultrasound.com.

3 www.vermon.com.

4 www.ndigital.com.
